# Oxygen Supplementation Improves Oxygen Uptake Kinetics and Exercise Performance in PAH and CTEPH Patients

**DOI:** 10.1002/cph4.70194

**Published:** 2026-06-11

**Authors:** Shir Kadosh, Yael Baidats, Andrew M. Jones, Daryl Wilkerson, Ariela Velner, Ronen Reuveny, Michael J. Segel

**Affiliations:** ^1^ School of Public Health, Gray Faculty of Medicine Tel Aviv University Tel‐Aviv Israel; ^2^ Pulmonary Institute Sheba Tel HaShomer Medical Center Ramat Gan Israel; ^3^ Sport and Health Sciences, College of Life and Environmental Sciences University of Exeter Exeter UK; ^4^ Department of Physical Therapy, Faculty of Social Welfare and Health Sciences University of Haifa Haifa Israel; ^5^ Dina Recanati School of Medicine Reichman University Herzliya Israel; ^6^ Department of Medicine, Gray Faculty of Medicine Tel Aviv University Tel‐Aviv Israel

**Keywords:** CPET, exercise physiology, O_2_ supplementation, pulmonary vascular disease, V̇O_2_ kinetics

## Abstract

**Aim:**

We studied the effect of O_2_ supplementation on physiological responses to exercise in patients with pulmonary vascular disease.

**Methods:**

Six patients with pulmonary arterial hypertension (PAH), four patients with chronic thromboembolic pulmonary hypertension (CTEPH) (age 54 ± 17 years; 8 females) and 13 healthy individuals (age 50 ± 17 years; 5 females) were tested. PAH/CTEPH was defined hemodynamically by mPAP > 20 mmHg and PVR > 3 WU. Patients performed symptom‐limited cardiopulmonary exercise tests, and constant work‐rate tests (CWRTs) at 80% of the work‐rate (WR) at the gas exchange threshold (GET). Tests breathing room air (RA, 21% O_2_) were compared to tests performed breathing 30% O_2_. Oxygen‐uptake (V̇O_2_) kinetics were calculated from the CWRT results.

**Results:**

In the PAH/CTEPH group, peak WR, peak V̇O_2_ and V̇O_2_ at the GET improved significantly when breathing 30% O_2_ compared to RA (mean ± SD 85 ± 26 vs. 77 ± 25 W, 18.3 ± 5.8 vs. 15.6 ± 5.7 mL/kg/min and 764 ± 181 vs. 685 ± 154 mL/min; *p* = 0.011, *p* = 0.015 and *p* = 0.012, respectively). Peak V̇O_2/_HR was higher with 30% O_2_ compared with RA (8.8 ± 2.2 vs. 7.7 ± 1.8 mL/beat, *p* = 0.021). The time constant (tau) of V̇O_2_ kinetics was faster in PAH/CTEPH patients while breathing 30% O_2_ compared to RA (36 ± 4 vs. 43 ± 6 s, *p* = 0.009). In healthy individuals there was no improvement in V̇O_2_ kinetics while breathing 30% O_2_ compared to RA (tau 35 ± 6 vs. 35 ± 6 s, *p* = 0.916).

**Conclusion:**

A clinically applicable level of O_2_ supplementation (30%) improved maximal and aerobic exercise capacity and V̇O_2_ kinetics in PAH/CTEPH patients. O_2_ supplementation may be considered to support exercise training in PAH/CTEPH patients.

## Graphical abstract

In patients with pulmonary vascular diseases (PAH and CTEPH), supplemental oxygen (FiO_2_ 0.3), compared to room air (FiO_2_ 0.21) improved peak exercise capacity (peak V̇O_2_), peak sustainable exercise (V̇O_2_ at GET), and V̇O_2_ kinetics and the onset of exercise.

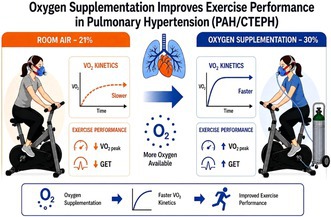

**Abbreviations**
BMI: body mass index
COPD: chronic obstructive pulmonary disease
CPET: cardiopulmonary exercise testing
CWRT: constant work‐rate tests
DLCO: carbon monoxide diffusion capacity
FEV_1_
: forced expiratory volume in one second
GET: gas exchange threshold
HR: heart rate
MVV: maximal voluntary ventilation
PhI: phase I of V̇O_2_ kinetics
PhII: phase II of V̇O_2_ kinetics
RER: respiratory exchange ratio
RV: residual volume
SpO_2_
: oxygen saturation
TLC: total lung capacity
V̇CO_2_
: carbon dioxide production
V̇_E_
: minute ventilation
V̇O_2_
: oxygen consumption
V_T_
: tidal volume
*τ*
: time constant of V̇O_2_ kinetics

## 1 Introduction

Pulmonary vascular diseases are a group of diseases characterized hemodynamically by pre‐capillary pulmonary hypertension (PH), defined by mean pulmonary artery pressure > 20 mmHg and normal left ventricular (LV) filling pressure (Humbert et al. 2022; Simonneau et al. 2019). There are two major forms of pulmonary hypertension that primarily affect the pulmonary vasculature: pulmonary arterial hypertension (PAH, group 1 PH) and chronic thromboembolic pulmonary hypertension (CTEPH, group 4 PH) (Humbert et al. 2022). In response to the excessive proliferation of endothelial, smooth muscle, and adventitial cells in pulmonary vessels (in PAH), or as a result of incomplete resolution of pulmonary embolism (in CTEPH), pulmonary vascular resistance (PVR) progressively increases, leading to right ventricular (RV) cardiac failure and premature death. Typical symptoms include effort dyspnea and leg fatigue, causing exercise intolerance and reduced quality of life.

Oxygen (O_2_) supplementation is not commonly prescribed to enhance exercise tolerance in non‐hypoxemic patients with PAH/CTEPH, though several studies indicate potential benefits. Ulrich et al. demonstrated that O_2_ supplementation (FiO_2_ = 0.5) improved peak work rate (WR) by 16% and endurance time by 117% (Ulrich et al. 2017). In another study, the same group reported that 5 weeks of domiciliary O_2_ therapy led to significant improvements in exercise performance, quality of life, and New York Heart Association Classification (NYHA) functional class in 30 PAH/CTEPH patients with resting SpO2 ≥ 90% and exercise‐induced hypoxemia, defined as a ≥ 4% drop to ≤ 92% during a six‐minute work test on room air (RA) (Ulrich et al. 2019).

Boutou et al. observed increased exercise performance, cardiac output, and cerebral oxygenation with hyperoxia (FiO_2_ = 0.4) in nine PAH/CTEPH patients without resting hypoxemia (PaO_2_ ≥ 60 mmHg/SpO_2_ ≥ 91%) but with desaturation during exercise (≥ 4% SpO_2_ drop compared to resting levels) (Boutou et al. 2021).

The mechanisms underlying the acute improvement in exercise tolerance with O_2_ supplementation in PAH/CTEPH patients remain to be elucidated (Jones and Poole 2005). Studying oxygen uptake (V̇O_2_) kinetics provides insights into muscle oxidative metabolism. V̇O_2_ kinetics during moderate‐intensity constant work rate tests (CWRTs) performed below the gas exchange threshold (GET) have been suggested to more accurately reflect patient's daily activities compared to maximal (symptom‐limited) ramp exercise tests (Jones and Poole 2005). V̇O_2_ kinetics following the onset of submaximal exercise can objectively assess aerobic capacity and provide insight into possible physiological limitations to oxygen transport and utilization in active muscles. The kinetics of V̇O_2_ after the onset of exercise can be divided into three phases: Phase I reflects the immediate increase in pulmonary blood flow (15–20 s); Phase II is the exponential increase in V̇O_2_ in the exercising muscle until a steady state is reached; and Phase III reflects the steady‐state level of oxygen consumption by working muscles (Jones and Burnley 2009). One study (Sietsema 1992) found that patients with PAH/CTEPH had slower V̇O_2_ kinetics (i.e., longer mean response time [MRT]) and a correspondingly larger O_2_ deficit compared to healthy controls. We studied how a clinically applicable level of O_2_ supplementation (30% O_2_) affects V̇O_2_ kinetics and exercise tolerance in PAH/CTEPH patients who are not hypoxemic. We hypothesized that 30% O_2_ would speed V̇O_2_ kinetics and improve exercise capacity.

## 2 Methods

### 2.1 Subjects

The study was approved by the Sheba Medical Center Institutional Review Board (No. 8242‐21 SMC). All subjects provided written informed consent. The PAH/CTEPH group included patients with PAH or CTEPH diagnosed according to international guidelines (Humbert et al. 2022), including confirmation of pre‐capillary pulmonary hypertension by right heart catheterization. CTEPH patients had either inoperable, distal disease or significant residual CTEPH after pulmonary endarterectomy surgery and/or balloon pulmonary angioplasty. PAH/CTEPH patients were of WHO functional capacity I or II. Patients in groups 2, 3, or 5 in the clinical classification of pulmonary hypertension, patients with a pacemaker, any heart block, history of life‐threatening arrhythmia, or syncope on exertion or oxygen saturation at rest < 85% were excluded. The healthy group consisted of healthy volunteers without any significant disorder expected to affect physiological responses to exercise (Baidats et al. 2024).

### 2.2 Experimental Design

The design was a single‐blind random‐order crossover study. PAH/CTEPH patients attended the exercise laboratory on four separate occasions. They were instructed to take their medication as usual and to have a light breakfast before the tests. Each PAH/CTEPH patient completed two symptom‐limited ramp cardiopulmonary exercise tests and eight CWRTs (see further explanations of the CWRTs below) while breathing RA (FiO_2_ = 0.21) or 30% O_2_ (FiO_2_ = 0.30), a clinically applicable concentration of O_2_. The test sequences for RA and 30% O_2_ were determined by the last digit of the birth year. Participants were unaware of the FiO_2_ of each individual test. During the first visit, spirometry, measurement of lung volumes by body plethysmography, and assessment of single‐breath carbon monoxide diffusion capacity (D_L_CO) were performed (Masterscreen PFT Pro, Jaeger, Germany). Maximal (symptom‐limited) exercise tests were performed on visits 1 and 2. During visits 3 and 4, PAH/CTEPH patients performed a total of eight CWRTs at 80% of the WR at the GET. In each of visits 3 and 4, two tests were performed per condition, in random order. Spirometry was repeated on each visit to ensure stability between the four study days.

Healthy participants attended the laboratory three times. They performed a maximal exercise test with only RA (visit 1), and eight CWRTs, four per condition (RA and 30% O_2_; visits 2 and 3). The entire sequence of tests for each individual in both groups was completed within 30 days.

### 2.3 Intervention: Supplemental O_2_

Supplemental O_2_ was administered as previously described (Baidats et al. 2024). Inspired air was delivered by a 200 L meteorological balloon, which served as a reservoir bag, connected to a gas‐mixing blender (NEO_2_ BLEND, Bio‐Med Devices Inc., Guilford, CT, USA). The reservoir was connected by a corrugated tube to a Hans Rudolph 2700 two‐way non‐rebreathing valve to ensure entry of O_2_‐enriched air into the metabolic face mask (without mixing with RA) and prevented the entry of exhaled air back into the balloon. A flow sensor (Cosmed, Italy) was connected to the mask. The flowmeter turbine was calibrated using a 3 L syringe, while *gas calibration* was completed *with* a *16*% *O*_*2*_ and 5% *CO*_*2*_ standard prior to each test. To ensure that equilibrium was achieved between the gas in the reservoir bag and alveolar gas, participants sat motionless on the cycle ergometer for 10 min before initiating the test, while breathing air from the reservoir (Ward et al. 2017). FiO_2_ was monitored throughout the tests by the metabolic system.

### 2.4 Symptom‐Limited Ramp Cardiopulmonary Exercise Tests

Incremental symptom‐limited exercise tests were performed as previously described (Baidats et al. 2024) on an electronically braked cycle ergometer (Ergoselect 600, Ergoline, Cosmed, Italy). The participant breathed through a face mask (V2mask, Hans Rudolph Inc., Shawnee, KS, USA) connected to a metabolic cart (Quark CPET, Cosmed, Italy). Exercise protocols were designed to ensure that, following 3 min of rest and 3 min of unloaded cycling, participants reached volitional exhaustion within 8–12 min of incremental exercise: The WR increment ranged from 5 to 25 watts/min depending on predicted peak V̇O_2_ adjusted for estimated individual fitness level, based on symptoms and pulmonary function tests (Hansen et al. 1984) The required WR increment was calculated, assuming V̇O_2_ of 500 mL/min during unloaded cycling and ΔV̇O_2_:ΔWR of 10 mL/min/W, as follows: WR increment (W/min) = (adjusted predicted peak V̇O_2_ (mL/min) – 500)/100.

Respiratory and metabolic measures, HR, 12‐lead electrocardiogram (Norav Medical Ltd., Mainz‐Kastel, Germany) and SpO_2_% were continuously monitored and recorded throughout the test. The measured variables were assessed breath‐by‐breath and presented for evaluation as mean values at 10 s intervals. At the end of each test, dyspnea and leg fatigue were rated by participants according to the modified Borg scale (rated from 1 to 10) (Borg 1982). Peak V̇O_2_ was determined by averaging the two highest consecutive 20 s values. GET was determined visually by two researchers using the V‐slope method (Wasserman 1987). V̇CO_2_ was plotted against V̇O_2_. Two distinct linear slopes were then identified: an initial slope representing aerobic metabolism, and a steeper upper slope reflecting the added CO_2_ from lactate buffering. GET was determined to be V̇O2 at the intersection of the two slopes. If the difference between the two evaluators was greater than 5%, the threshold was determined by consensus in consultation with a third researcher. Maximal voluntary ventilation (MVV, in L/min) was estimated as 40 times the measured forced expiratory volume in 1 s (FEV_1_) (Hansen et al. 1984).

### 2.5 Constant Work Rate Tests

In order to measure V̇O_2_ kinetics, participants performed CWRTs as previously described (Baidats et al. 2024). In order to reduce variability and enhance the signal‐to‐noise ratio, each subject performed 4 repeat transitions for each condition (RA and 30% O_2_) (Sietsema 1992), from complete rest to moderate‐intensity exercise at a WR of 80% of the lower GET as determined from the two maximal effort tests (Barstow et al. 1990; Casaburi et al. 1989). In 2 participants (1 PAH/CTEPH, 1 healthy) in whom GET was not achieved or was not clearly determined in either of the maximal tests, CWRTs were performed at a work rate equal to 40% of the maximum work rate achieved. Participants sat motionless on the cycle ergometer for 3 min before initiating exercise while breathing RA or 30% O_2_, then started pedaling at a rate of 65 rpm for 6 min (Casaburi et al. 1989). During these tests, ventilatory and gas exchange responses were recorded. Participants were asked to rate dyspnea and leg fatigue according to the Borg scale after completing each CWRT. Adequate rest (at least 15 min) was provided between tests so that metabolism (as inferred from heart rate and ventilatory rate) returned to resting levels. Variables including plateau end‐tidal partial pressure of carbon dioxide (P_ET_CO_2_), minute ventilation (V̇_E_), respiratory exchange ratio (RER), and ventilatory equivalent for O_2_ (V̇_E_/ V̇O_2_) were calculated as the mean of the last 60 s of four CWRTs for each condition.

### 2.6 Oxygen Uptake Kinetics Modeling

V̇O_2_ kinetics data were analyzed as previously described (Jones and Burnley 2009; Baidats et al. 2024; Wilkerson et al. 2006). Breath‐by‐breath V̇O_2_ measurements display considerable inherent variability (i.e., “noise”) and outliers are often present (errant breaths caused by coughing, swallowing, premature ending of a breath, etc.). Therefore, prior to analysis, raw data from each test were examined to exclude data that inappropriately reflected the underlying physiology (e.g., points lying more than 4 standard deviations from the 5‐breath local rolling mean). Once this editing process was complete, data from each test were linearly interpolated to second‐by‐second values. This enabled breath‐by‐breath data from the four tests to be time‐aligned and ensemble‐averaged, to further improve the signal‐to‐noise ratio (Barstow et al. 1990). The basal V̇O_2_ value (resting V̇O_2_) was determined by averaging data obtained during the last 60 s of the rest period. The phase I to phase II transition (Ph‐I‐II‐tr.) was visually determined by identifying the point at which a sharp decrease from the baseline value occurred in the RER (Sietsema 1992; Mezzani 2010). This point is usually associated with an inflection in the rate of rise of V̇O_2_. When the beginning of the decrease in RER was not sufficiently clear, the end of phase I was identified from the V̇O_2_ inflection point alone. Phase II data were imported into a purpose‐written Excel‐based modeling program that fits the V̇O_2_ response using a nonlinear mono‐exponential least‐square regression algorithm in accordance with the following equation:

$$\dot{V}O_{2}\;\left(t\right)=\dot{V}O_{2}\text{baseline}+A\;\left(1-e^{-\left(t-\mathrm{TD}\right)/\tau }\right)$$

where V̇O_2_ (t) represents the absolute V̇O_2_ at any given time t; V̇O_2_ baseline, the mean V̇O_2_ at rest; A, the steady‐state amplitude of the V̇O_2_ response; TD, the time delay, equal to the individually determined phase I duration; and *τ*—the time constant of the phase II increase in V̇O_2_. The model was fitted to the V̇O_2_ data of each subject using a time window starting from directly determined Ph‐I‐II‐tr.

### 2.7 Data Analysis and Statistical Methods

We conducted a preliminary sample size estimation using WinPepi software (Erdfelder et al. 2009) to achieve a significant difference in peak V̇O_2_ and V̇O_2_ kinetics within participants from the same group (patients/healthy) when using 21% O_2_ compared to 30% O_2_ and a significant difference between the groups themselves at the desired power. Following a previous study on the impact of O_2_ supplementation on the V̇O_2_ kinetics of COPD patients (Palange et al. 1995), which showed a substantial effect of 30% O_2_ (effect size = 0.85), we conservatively chose an effect size of 0.75. The minimum required sample size to achieve 80% power was calculated to be 8 participants in each group.

Age, body weight, height, body mass index (BMI) and pulmonary function test results are reported as mean ± SD. Due to the small sample size, we conservatively used non‐parametric statistical tests that do not assume normal distribution. The Wilcoxon signed‐rank test was used to compare paired individual values between the two conditions (RA and 30% O_2_). The time constant *τ* was compared between groups (patients vs. healthy) using the Mann–Whitney *U* test. Pearson's test was used to assess correlation between variables. A *p*‐value < 0.05 was considered statistically significant. All data were analyzed using IBM SPSS statistical software (version 28.0.1.0).

## 3 Results

**FIGURE 1:**
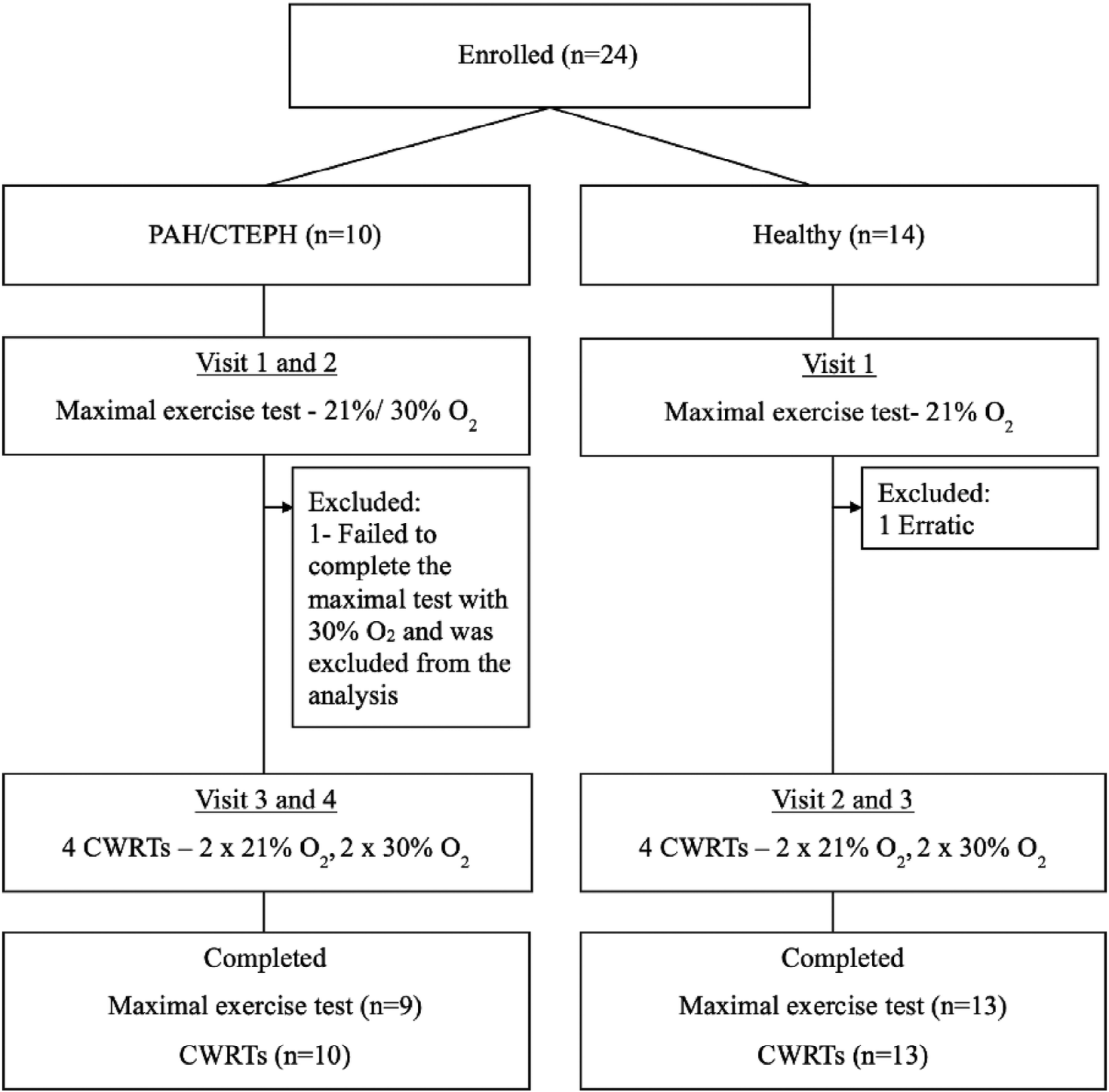
Flowchart of participants in the study.

Twenty‐four participants (10 PAH/CTEPH, 14 healthy) were enrolled in the study (Figure 1). One subject from the PAH/CTEPH group failed to complete the maximal test with 30% O_2_. One participant from the healthy group was excluded from the analysis due to highly erratic breathing. Descriptive data, including subject characteristics and lung function test results, are presented in Table 1. The two groups were well matched for age, height, body weight, body mass index (BMI), and SpO_2_% at rest. There was no significant difference in FEV_1_% predicted between the groups (86% ± 21% vs. 96% ± 15%, *p* = 0.37). D_L_CO was significantly lower in the PAH/CTEPH patients compared with the healthy group (66% ± 19% vs. 91% ± 13%, *p* = 0.003).

**TABLE 1:** Characteristics and lung function of PAH/CTEPH and healthy controls.

	PAH/CTEPH	Healthy	*p*‐value
*N* (female)	10 (8)	13 (6)	
Age	54 ± 17	50 ± 17	NS
Height	166 ± 7	172 ± 12	NS
Weight	71 ± 7	75 ± 18	NS
Body‐mass index, kg/m^2^	26 ± 3	25 ± 4	NS
WHO functional class	I (40%)		
	II (60%)		
6‐min walk distance, m	485 ± 69		
Classification			
Heritable PAH	1 (80%)		
Idiopathic PAH	4 (40%)		
CTD‐PAH (Scleroderma)	1 (80%)		
Chronic thromboembolic pulmonary hypertension	4 (40%)		
SpO_2_% at rest	99 ± 0.8	100 ± 1	0.088
Hemodynamics			
Mean pulmonary artery pressure (mmHg)	36 ± 11		
Right atrial pressure (mmHg)	6 ± 5		
Pulmonary vascular resistance (WU)	6 ± 4		
HR rest (beat/min)	72 ± 11	66 ± 6	
Cardiac Index (L/min/m^2^)	3.0 ± 1.0		
Treatment			
Endothelin receptor antagonist	6 (60%)		
Phosphodiesterase‐5‐inhibitor	5 (50%)		
Riociguat	3 (30%)		
Prostanoid	4 (40%)		
Sotatercept	2 (20%)		
Lung function			
Forced expiratory volume in 1 s (FEV1), % predicted	86 ± 21	98 ± 13	NS
Forced vital capacity (FVC), % predicted	95 ± 17	96 ± 14	NS
FEV1/FVC	76 ± 13	83 ± 7	NS
Total lung capacity, % predicted	107 ± 14	98 ± 12	NS
Diffusion capacity for carbon dioxide, % predicted	66 ± 19	92 ± 16	0.002

*Note:* Values are average (±SD); A value of *p* < 0.05 was considered as statistically significant; Munn‐Whitney Test for independents non‐parametric samples.

Abbreviations: BMI, body mass index; FEV1, forced expiratory volume in 1; FVC, forced vital capacity; HR, heart rate; PAH, pulmonary arterial hypertension; SpO_2_, oxyhemoglobin saturation.

### 3.1 Symptom‐Limited Ramp Exercise Tests

**FIGURE 2:**
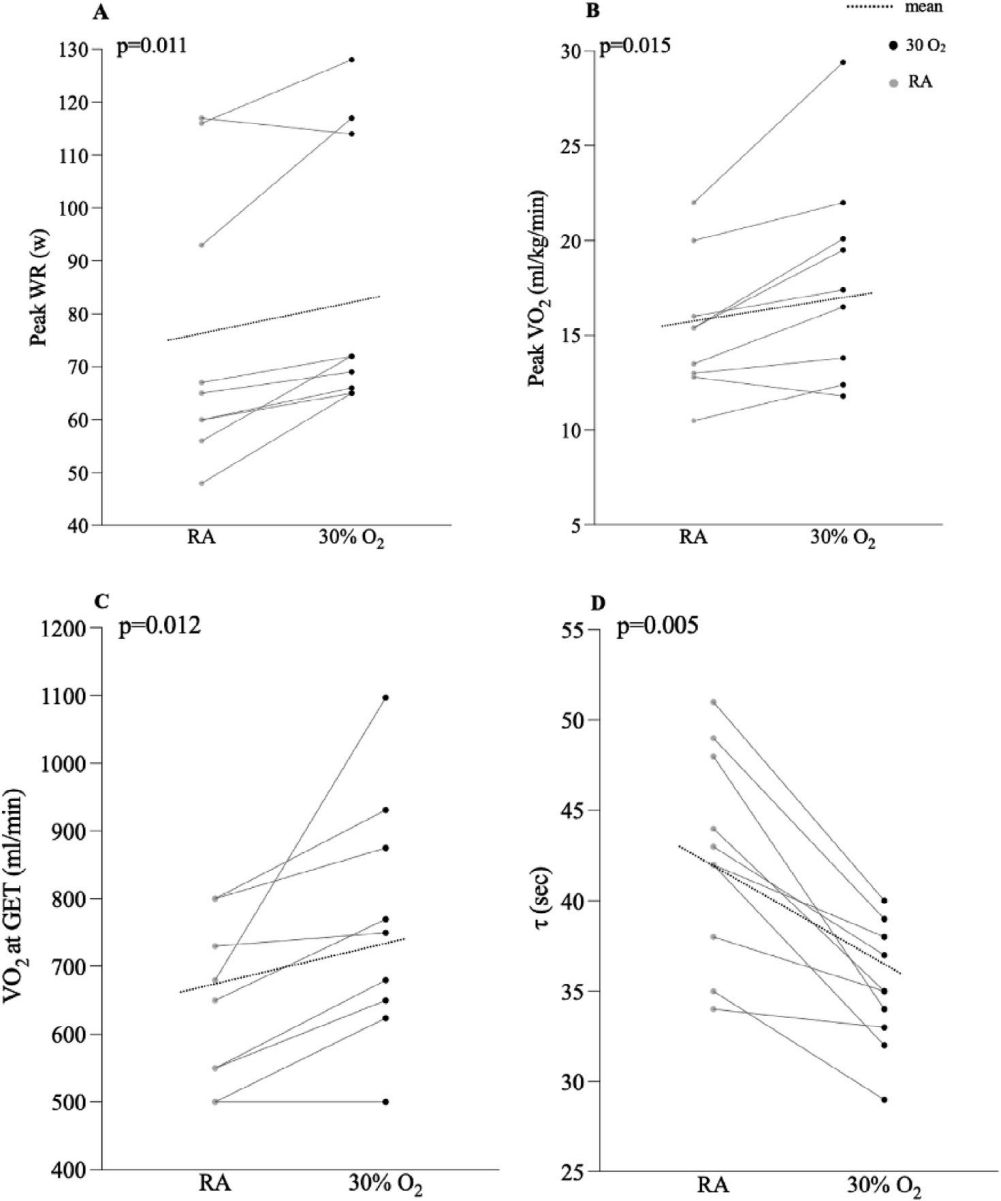
A comparison of physiological and performance variables during RA breathing and 30% O_2_ in patients with PAH/CTEPH: (A) peak V̇O_2_, (B) peak WR, (C) V̇O_2_ at GET, and (D) time constant (*τ*) of V̇O_2_ kinetics. Data points represent results from individual participants. Dashed line represents mean values.

**FIGURE 3:**
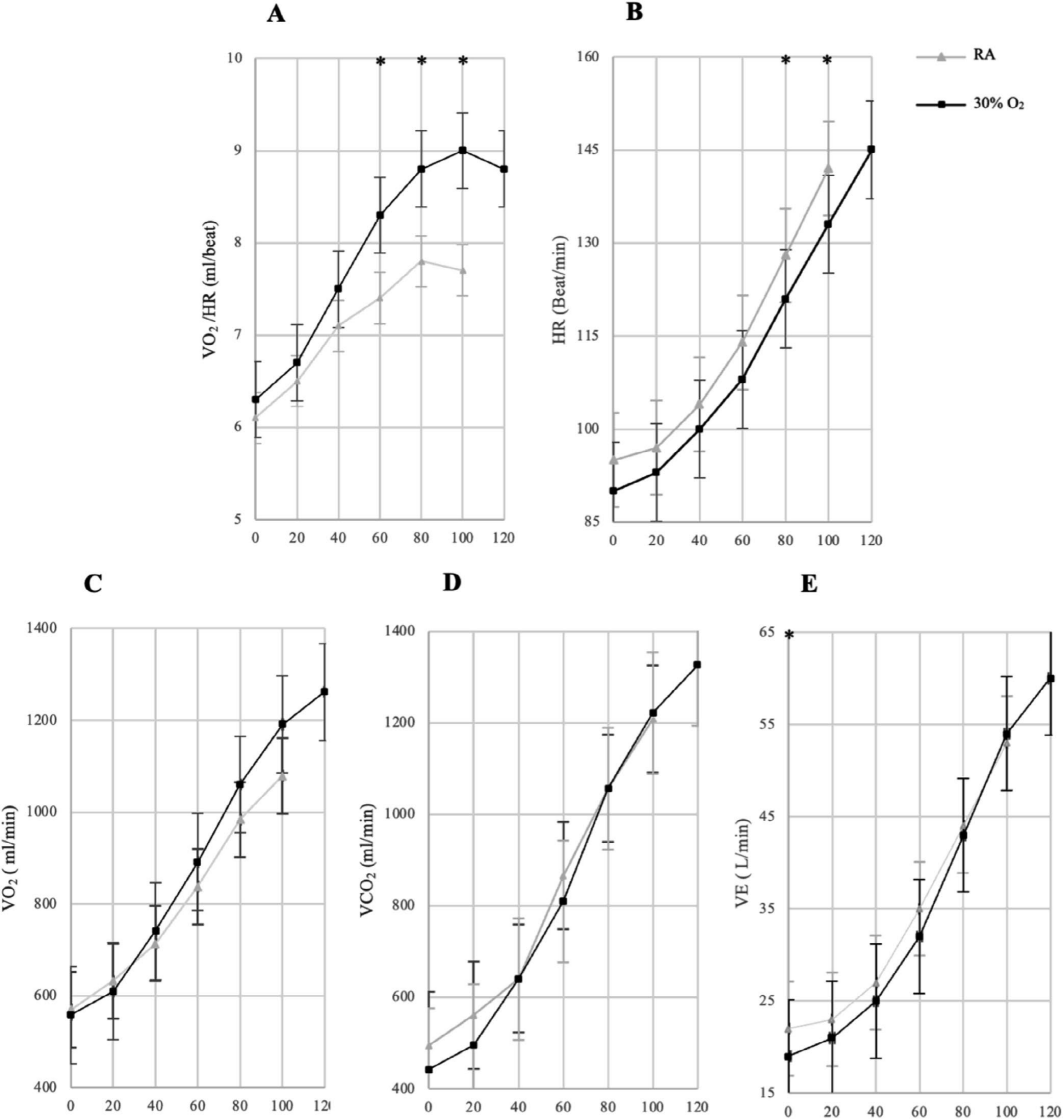
A comparison of (A) oxygen uptake/heart rate (V̇O_2_/HR), (B) heart rate (HR), (C) V̇O_2_, (D) carbon dioxide output (V̇CO_2_), (E) minute ventilation (V̇_E_) V̇O_2_ versus WR expressed as a percentage of the peak WR achieved during the RA tests, in tests breathing RA (gray triangles) and 30% O_2_ (black squares) in PAH/CTEPH patients. All values are mean ± SD.

The results of the ramp exercise tests are summarized in Table 2. As expected, compared to the healthy group, the PAH/CTEPH group had lower peak V̇O_2_, lower peak WR and lower V̇O_2_ at the GET. PAH/CTEPH subjects significantly increased exercise capacity when breathing 30% O_2_ compared to RA. Peak WR increased with RA compared to 30% O_2_ (*p* = 0.011) and peak V̇O_2_ increased from 15.6 ± 5.7 mL/kg/min with RA to 18.3 ± 5.8 mL/kg/min with 30% O_2_ (*p* = 0.015; Figure 2A,B). V̇O_2_ at the GET increased from 685 ± 154 mL/min with RA to 764 ± 181 mL/min with 30% O_2_ (*p* = 0.012) (Figure 2C). In addition, peak V̇O_2/_HR and ∆V̇O_2_/∆WR were higher with 30% O_2_ compared with RA (*p* = 0.021 for both); O_2_ saturation improved slightly but significantly with 30% O_2_ (99% ± 1% vs. 96% ± 3%, *p* = 0.007). Peak heart rate, peak V̇_E_, and Borg scale dyspnea score were unaffected by 30% O_2_, suggesting that the participants' degree of exertion was similar. A non‐significant trend to improvement was observed with 30% O_2_ in the ventilatory equivalents for oxygen (V̇_E_/V̇O_2_) and carbon dioxide (V̇_E_/V̇CO_2_) at GET (*p* = 0.107, respectively), suggesting gas exchange may be more efficient with 30% O_2_, but this interpretation should be made cautiously given the non‐significant results. Figure 3 illustrates the physiological responses of PAH/CTEPH patients to exercise, expressed as a percentage of the peak WR attained while breathing RA. The difference in V̇O_2_/HR at iso‐WR between conditions gradually increased, becoming significant from 60% of the peak WR to the end of the test (Figure 3A). HR at iso‐time was uniformly lower with 30% O_2_ (Figure 3B). V̇O_2_ at iso‐time, higher with 30% O_2_, began to diverge around 40% of the peak‐WR (RA), becoming statistically significant at 100% of iso‐work (Figure 3C). Carbon dioxide output (V̇CO_2_) followed a similar pattern in both conditions (Figure 3D). V̇E was significantly lower at rest during the 30% condition, with no differences observed between the two conditions from 20% to 100% iso‐WR. However, at peak exercise, V̇_E_ was higher with 30% O_2_ compared to RA (Figure 3E).

**TABLE 2:** Physiological responses‐Incremental symptom‐limited exercise tests.

	PAH/CTEPH			Healthy	*p*‐value^2^
	RA	30% O_2_	*p*‐value^1^	RA	
Peak WR (W)	77 ± 25	85 ± 26	0.011	149 ± 49	0.001
Peak V̇O_2_ (mL/kg/min)	15.6 ± 5.7	18.3 ± 5.8	0.015	22.8 ± 5.8	0.001
Peak V̇O_2_ (% predicted)	68 ± 15	78 ± 16	0.01	83 ± 13	< 0.001
Peak V̇O_2_/HR (mL/beat)	7.7 ± 1.8	8.8 ± 2.2	0.021	11.4 ± 4	0.0**2**1
Heart rate_peak_ (beat/min)	142 ± 18	145 ± 20	NS	152 ± 26	NS
HRR min^−1^	28 ± 18	22 ± 13	0.171	18 ± 13	0.006
∆V̇O_2_/∆WR (mL/min/W)	6.5 ± 1.5	8.1 ± 1.2	0.021	8.2 ± 1.2	0.001
V̇O_2_ at GET (mL/min)	685 ± 154	764 ± 181	0.012	985 ± 222	< 0.001
WR at GET (W)	32 ± 15	45 ± 13	0.021	67 ± 21	< 0.001
RER at peak exercise	1.13 ± 0.13	1.06 ± 0.08	0.05	1.25 ± 0.75	< 0.001
Peak V̇_E_ (L/min)	53 ± 10	60 ± 23	NS	74 ± 21	0.014
V̇_E_/MVV ratio (%)	61 ± 13	66 ± 12	NS	61 ± 17	NS
V̇_E_/V̇CO_2_ at GET	40 ± 3	38 ± 3	NS	31 ± 5	< 0.001
V̇_E_/V̇O_2_ at GET	38 ± 4	36 ± 5	NS	29 ± 6	< 0.001
S_P_O_2_% at peak	96 ± 3	99 ± 1	0.007	99 ± 1	< 0.001
Dyspnea (Borg)	5 ± 1	5 ± 2	NS	5 ± 2	NS
Leg discomfort (Borg)	6 ± 2	5 ± 2	NS	6 ± 1	NS

*Note:* (1) RA, PAH/CTEPH vs. Healthy. (2) PAH/CTEPH, RA vs. 30% O_2_. Values are mean (± SD); *p* < 0.05 was considered as statistically significant.

Abbreviations: GET, gas exchange threshold; HR, heart rate; HRR, heart rate reserve; MVV, maximal voluntary ventilation; NS, non‐significant; RA, room air; SPO_2_, oxygen saturation by pulse oximetry; V̇CO_2_, carbon dioxide production; V̇_E_, minute ventilation; V̇O_2_, oxygen consumption; WR, work rate.

### 3.2 V̇O_2_ Kinetics

**FIGURE 4:**
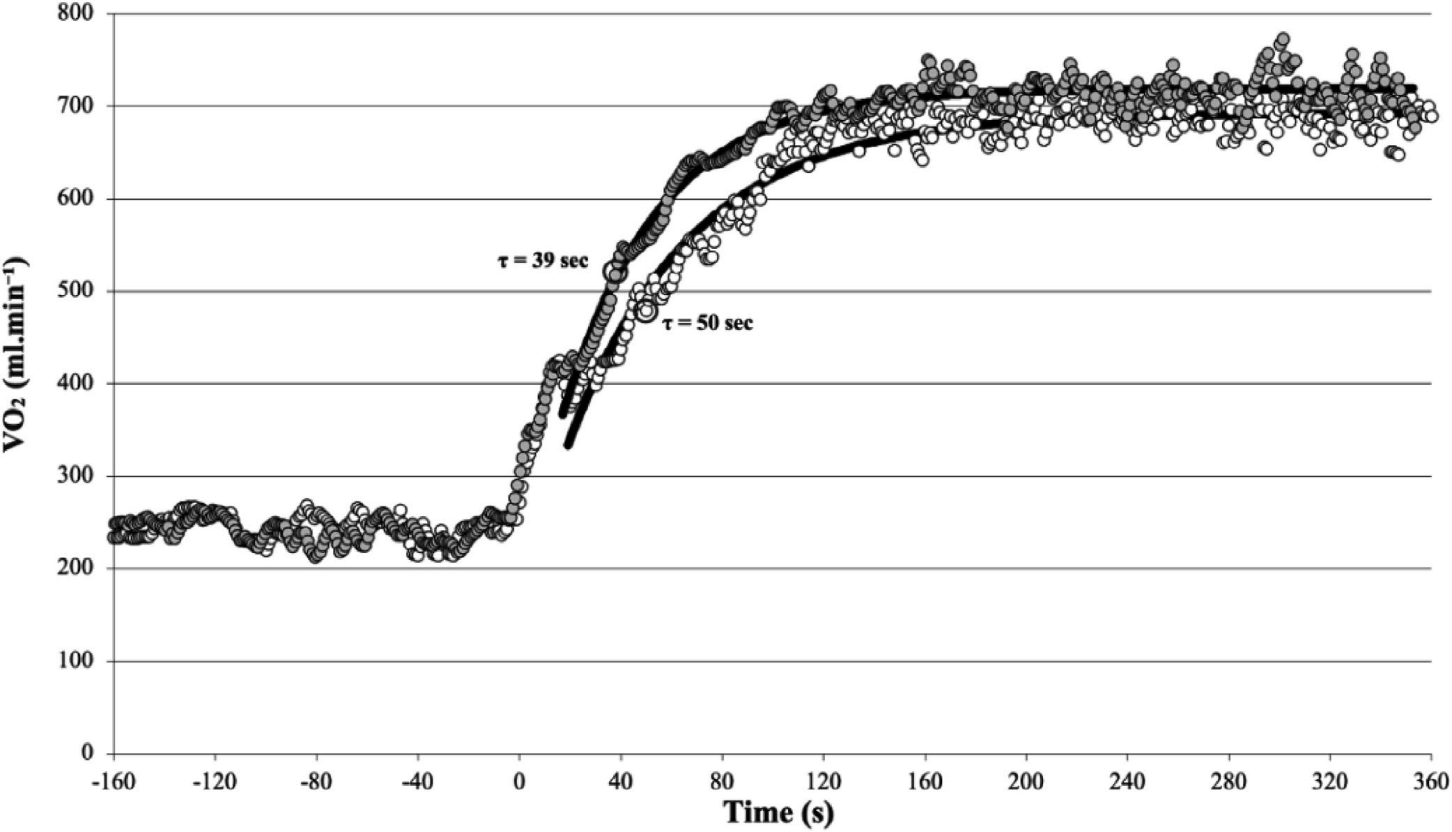
Oxygen uptake (V̇O_2_) as a function of time during the transition from rest to constant work‐rate moderate‐intensity exercise in a patient with pulmonary arterial hypertension (PAH) while breathing room air (RA; white circles) and 30% O_2_ (gray circles). Time 0 represents start of exercise. The time constant of Phase II V̇O_2_ kinetics (*τ*; black circles) was longer while breathing RA (*τ* = 50 s) compared to breathing 30% O_2_ (*τ* = 39 s).

Results of CWRTs, in which participants transitioned from rest to moderate intensity exercise (see details in Methods section), are shown in Table 3. In the PAH/CTEPH group, the time constant (τ) of phase II of V̇O_2_ kinetics (breathing RA) was slower than that in the healthy group (43 ± 6 vs. 35 ± 6 s, *p* < 0.005). Breathing 30% O_2_ had no effect on V̇O_2_ kinetics in the healthy participants but accelerated V̇O_2_ kinetics in the PAH/CTEPH group (Figure 2D) such that the time constant was the same as that of the healthy group (Table 3). The V̇O_2_ kinetics of a representative PAH/CTEPH patient are illustrated in Figure 4, showing faster kinetics (lower τ) while breathing 30% O_2_ (39 s) compared to RA (50 s). The O_2_ deficit was lower while breathing 30% O_2_ compared to RA. As expected, S_P_O_2_% during CWRTs was slightly higher with 30% O_2_ compared to RA (99 ± 1 vs. 97% ± 4%, *p* = 0.001). In the PAH/CTEPH group, supplemental O_2_ slightly reduced steady‐state (phase III) V̇_E_/V̇O_2_ and V̇_E_/V̇CO_2_ (37 ± 4 to 35 ± 4, *p* = 0.011, and 39 ± 4 to 38 ± 5, *p* = 0.048 respectively). This reduction was driven by lower steady‐state ventilation (V̇_E_) (*p* = 0.009) and was associated with higher steady‐state end‐tidal CO_2_ (P_E_TCO_2_).

**TABLE 3:** Physiological responses to constant work rate tests.

	PAH/CTEPH			Healthy		
	RA	30% O_2_	*p*‐value	RA	30% O_2_	*p*‐value
V̇O_2_ baseline (mL/min)	212 ± 58	213 ± 50	NS	295 ± 58	288 ± 52	NS
WR (W)	26 ± 10			49 ± 15		
WR %peak (W)	33 ± 6			33 ± 4		
Steady state V̇O_2_ (mL/min)	766 ± 119	769 ± 126	NS	1074 ± 237	1079 ± 288	NS
Steady state V̇CO_2_ (mL/min)	690 ± 109	695 ± 98	NS			
*τ* (s)	43 ± 6	35 ± 3	0.005	35 ± 6	35 ± 6	NS
O_2_ deficit (L)	0.47 ± 0.12	0.42 ± 0.11	0.019	0.57 ± 0.15	0.61 ± 0.18	NS
Steady state HR min^−1^	104 ± 17	100 ± 16	0.02			
Steady state V̇O_2_/HR (mL/beat)	7.7 ± 2.1	8.0 ± 2.2	0.007			
Steady state V̇_E_/V̇CO_2_	39 ± 4	38 ± 5	0.048			
Steady state V̇_E_/V̇O2	37 ± 4	35 ± 4	0.011			
Steady state P_E_TCO_2_ (mmHg)	28 ± 3	30 ± 3	0.016			
Steady state V̇_E_ (L/min)	30 ± 5	28 ± 4	0.009			
Steady state S_P_O_2_%	97 ± 3	99 ± 1	0.0011	99 ± 1	100 ± 1	NS
Dyspnea (Borg scale)	3 ± 1.3	2.5 ± 1.8	NS	2 ± 0.7	1.8 ± 0.7	NS
Leg discomfort (Borg scale)	3.1 ± 2.1	2.5 ± 2.6	NS	2.2 ± 0.9	2 ± 0.8	NS

*Note:* Values are average (± SD); A value of *p* < 0.05 was considered as statistically significant.

Abbreviations: *τ*, time constant; HR, heart rate; NS, non‐statistically significant; P_E_TCO_2_, end‐tidal partial pressure of carbon dioxide; PhI, phase I; RA, room air; SPO_2_, oxygen saturation by pulse oximetry; V̇CO_2_, carbon dioxide production; V̇_E_, minute ventilation; V̇O_2_, oxygen consumption; WR, work rate.

## 4 Discussion

We investigated the effect of O_2_ supplementation on exercise capacity in PAH/CTEPH patients with normal SpO_2_ at rest and during exertion. Our key findings were: 1. A clinically applicable level of supplemental O_2_ (30% O_2_) enhanced peak exercise and aerobic power (peak WR and peak V̇O_2_) and sustainable exercise capacity (reflected by V̇O_2_ at GET) in PAH/CTEPH patients. 2. V̇O_2_ kinetics were slower in PAH/CTEPH patients than in healthy individuals while breathing RA. 3. Although PAH/CTEPH patients were not hypoxemic during exercise (SpO_2_ 96%–99%), supplemental O_2_ accelerated V̇O_2_ kinetics and reduced the O_2_ deficit. The speeding of V̇O_2_ kinetics can perhaps be intuited to represent a more rapid “acceleration time” of muscle oxidative metabolism following the onset of exercise in patients receiving supplemental O_2_.

Ours is the first study in PAH/CTEPH patients to evaluate the effect of O_2_ supplementation on exercise metabolism and gas exchange. Moreover, we studied patients who were normoxemic during exercise (SpO_2_ 96%–99%). Our findings therefore suggest that supplemental O_2_ may play a significant role in improving exercise performance in PAH/CTEPH patients, even those without hypoxemia at rest or during exertion.

### 4.1 Effect of Hyperoxia on Maximal Exercise Performance in PAH/CTEPH Patients

Ulrich et al. (2017) demonstrated that O_2_ supplementation (FiO_2_ 0.5) significantly improved peak WR in hypoxemic PAH/CTEPH patients (NYHA 2‐3). This improvement was associated with better arterial, muscular, and cerebral oxygenation, reduced sympathetic activation, and improved pulmonary gas exchange. The performance benefits were linked to a higher PaCO_2_ at peak exercise and a lower V̇_E_/V̇CO_2_ ratio, suggesting that O_2_‐induced pulmonary vasodilation played a role in the improvement. Expanding on these results, we measured the effect of O_2_ supplementation on exercise gas exchange and observed significant increases in peak V̇O_2_, peak V̇O_2_/HR, V̇O_2_ at GET, peak WR, and ΔV̇O_2_/ΔWR. In hypoxemic patients, the mechanism likely involves an increase in arterial blood O_2_ content (higher SpO_2_), which enhances O_2_ delivery to muscles for aerobic energy production. In our study, the PAH/CTEPH patients maintained normal SpO_2_ levels at rest and exercise even when breathing RA. While breathing 30% O_2_ slightly increased exercise SpO_2_ (from 96% to 99%), it is unlikely that this small effect explains the entire benefit of O_2_ supplementation in our patients. We propose that O_2_ may additionally act as a pulmonary vasodilator, thus reducing right ventricular afterload, improving cardiac output and enhancing O_2_ delivery to muscle tissue. A recent study of acute vasodilator challenges during right heart catheterization in CTEPH found that oxygen (100%) caused a reduction of at least 20% in pulmonary vascular resistance (PVR) in 29% of the patients (Frantz et al. 2023). The major determinant of the pulmonary arterial tone's response to oxygen is alveolar PO_2_ (P_A_O_2_), with an additional contribution by arterial PO_2_ (P_a_O_2_) (Green and Stuart 2020). Both of these determinants would be modestly increased by 30% O_2_ supplementation. Green et al. (Green and Stuart 2020; Green and Stuart 2021) have proposed an alternative, endothelium‐dependent alveolar pathway. This theory suggests that an increase in alveolar O_2_ triggers a signaling cascade through endothelial cells, ultimately leading to vasodilation of pulmonary smooth muscle. This mechanism might explain pulmonary vasodilation even in non‐hypoxemic PAH/CTEPH patients such as those we studied, since the vasodilatory effect of O_2_ on pulmonary arterial tone appears to plateau at a PO_2_ of 100 mmHg (Sommer et al. 2008).

While we did not measure PVR in our study, two observations provide some indirect evidence that 30% O_2_ caused vasodilation in the PAH/CTEPH patients: (Humbert et al. 2022) Peak V̇O_2_/HR was higher with 30% O_2_, suggesting an increase in cardiac stroke volume (or in O_2_ extraction) during exercise, perhaps due to reduced cardiac afterload as a result of pulmonary vasodilation (Goulding et al. 2020; Simonneau et al. 2019). Steady state ventilatory equivalents were lower with 30% O_2_ in the CWRT (in the maximal tests there was a non‐significant trend to improvement). This improved gas exchange efficiency is presumably a result of vasodilatation, which reduces the ventilation‐perfusion ratio in affected lung units.

Another potential explanation for the benefit of oxygen supplementation we observed is an amelioration of RV ischemia. In PAH RV systolic pressure and wall stress increase, enhancing myocardial oxygen demand, whereas the coronary perfusion pressure generated by the LV does not change or even falls (Oknińska et al. 2024). This insult is likely to be particularly problematic during exercise. The beneficial effect of supplementation on RV myocardial oxygenation is supported by the, on average, 14% increase in V̇O_2_/HR at peak exercise that we observed in the PAH/CTEPH patients in the 30% O_2_ condition compared to RA. According to the Fick relationship, rearranged such that cardiac output is replaced by the product of stroke volume (SV) and arterio‐venous O_2_ content difference (Δa‐vC͠O_2_), V̇O_2_/HR represents the product of SV × Δa‐vC͠O_2_. By improving O_2_ delivery to the “O_2_ starved” RV, O_2_ supplementation is likely to improve SV.

### 4.2 Effect of Hyperoxia on V̇O_2_ Kinetics

The effects of hyperoxia on VO_2_ kinetics have been investigated across diverse populations, including healthy humans (Wilkerson et al. 2006), as well as patients with conditions such as COPD (Palange et al. 1995) and IPF (Baidats et al. 2024). Ours is the first study to examine the effects of supplemental O_2_ on V̇O_2_ kinetics in patients with pulmonary vascular disease (PAH/CTEPH). Phase II V̇O_2_ kinetics during the transition from rest to moderate exercise are believed to closely reflect the rate of O_2_ consumption in active muscles (Rossiter et al. 1999). Any alterations in O_2_ transport to the periphery or in O_2_ utilization by muscle mitochondria can affect V̇O_2_ kinetics (Goulding et al. 2020). V̇O_2_ kinetics provide valuable clinical insights into muscle energetics. Transitions between steady‐state energy levels frequently occur in daily life, and V̇O_2_ kinetics during constant work rate exercise at light‐to‐moderate intensity have been suggested as an accurate method for reflecting everyday activities in both healthy individuals and patients (Poole and Jones 2012; Alexander et al. 2003). Our results indicate that supplemental O_2_ improves V̇O_2_ kinetics in patients with pulmonary vascular disease. While the precise mechanisms by which supplemental O_2_ accelerates V̇O_2_ kinetics in non‐hypoxemic patients remain unclear, the benefits may arise from both central effects (as described earlier) and peripheral effects. A higher mean capillary PO_2_ increases the pressure gradient driving O_2_ diffusion into muscle cells, thus boosting O_2_ flux at the mitochondria (Wilkerson et al. 2006). This enables muscles to increase oxidative metabolism more rapidly, accelerating V̇O_2_ kinetics.

### 4.3 Effect of Supplemental O_2_ on V̇O_2_ Kinetics: Healthy Individuals vs. PAH/CTEPH Patients

Supplemental O_2_ did not improve V̇O_2_ kinetics in healthy individuals during moderate intensity exercise, consistent with previous studies (Wilkerson et al. 2006; MacDonald et al. 1997; Grassi et al. 1996) In contrast, healthy individuals show improvements in V̇O_2_ kinetics in response to aerobic exercise training. This might be understood to indicate that, in normal individuals, muscle capillary O_2_ delivery is not a limiting factor in V̇O_2_ kinetics. The effect of aerobic training on V̇O_2_ kinetics is likely mediated through an increase in capillary density, which enhances red blood cell mean transit time and diffusion area while reducing the diffusion distance from red blood cells to myocytes. Aerobic training also improves both the quality and quantity of mitochondria in the exercising muscle (Hellsten and Gliemann 2024). Conversely, in patients with PAH/CTEPH who suffer from a limited O_2_ supply to the muscles, O_2_ supplementation likely enhances O_2_ delivery to the exercising muscles, in part, through a pulmonary vasodilation mechanism, resulting in faster V̇O_2_ kinetics.

### 4.4 Limitations

A limitation of this study is its single‐blinded design—the investigators conducting the tests were aware of the O_2_ concentration given to the participants. As described in the Methods section, the metabolic cart was calibrated for the FiO_2_ used before every test, requiring the physiologists running the test to input the FiO_2_. Furthermore, during the tests, FiO_2_ measured breath by breath appears on the screen, which we considered very important to verify the assigned FiO_2_ was accurately delivered throughout the test. Thus, double blinding wasn't practical. However, the investigators provided consistent levels of encouragement across all conditions to minimize potential bias. Of note, in maximal exercise tests, there were no differences between RA and 30% O_2_ in the PAH/CTEPH group regarding peak HR, minute ventilation, Borg dyspnea score, or Borg leg discomfort score. This suggests that the absence of double blinding did not influence the participants' performance.

Another limitation is the small sample size. While the sample size was justified by our a priori calculation, we acknowledge that this should be regarded as an exploratory physiological study.

Finally, in our study we recruited only CTEPH/PAH patients with WHO functional class I and II symptoms. This was because the study was physically very demanding and we were concerned about dropout. We therefore acknowledge that our findings may not be generalizable to FC III and IV patients, although we tend to think the benefit of O_2_ supplementation would be the same or greater in sicker PH patients.

### 4.5 Clinical Implications

Although current guidelines do not recommend O_2_ supplementation for non‐hypoxemic patients (PaO_2_ > 8 kPa or SpO_2_ > 92%) (Humbert et al. 2022), largely based on evidence from COPD studies (Kawachi et al. 2021) our findings in normoxemic patients using a clinically applicable level of O_2_ supplementation (30%) as well as the work of Ulrich et al. (2019) suggest potential benefits. However, we acknowledge that ours is a small exploratory physiological study. Further research is needed to investigate the potential role of O_2_ supplementation during exercise training in PAH/CTEPH patients.

### 4.6 Conclusion

Oxygen supplementation (30% O_2_) enhanced maximal aerobic power (peak VO_2_), sustainable exercise capacity (GET), and VO_2_ on‐kinetics (“acceleration time”) during the transition from rest to moderate exercise in non‐hypoxemic patients with PAH/CTEPH. Supplemental O_2_ may reduce regional hypoxic pulmonary vasoconstriction, improving cardiac output and optimizing O_2_ delivery to exercising muscles. Therefore, O_2_ supplementation should be considered to support exercise training in PAH/CTEPH patients even in the absence of hypoxemia at rest or during exertion.

## Author Contributions

Conceptualization: S.K., R.R., M.J.S.; Methodology: S.K., Y.B., D.W., A.M.J., A.V., R.R., M.J.S.; Formal analysis: S.K., Y.B., D.W., R.R., M.J.S.; Data analysis: S.K., Y.B., D.W., R.R., M.J.S.; Visualization: S.K.; Original draft preparation: S.K., R.R., M.J.S.; Writing review and editing: S.K., Y.B., D.W., A.M.J., A.V., R.R., M.J.S.; Supervision and obtaining funds: R.R., M.J.S.

## Funding

This work was supported by the G. Baum Fund of the Israeli Lung Association, Tel‐Aviv.

## Disclosure

Some of the results herein were presented in abstract form at the European Sport Science Conference, Paris, France in July 2023.

## Conflicts of Interest

The authors declare no conflicts of interest.

## Data Availability

The data that support the findings of this study are available on request from the corresponding author. The data are not publicly available due to privacy or ethical restrictions.
